# Frataxin Silencing Inactivates Mitochondrial Complex I in NSC34 Motoneuronal Cells and Alters Glutathione Homeostasis

**DOI:** 10.3390/ijms15045789

**Published:** 2014-04-04

**Authors:** Barbara Carletti, Emanuela Piermarini, Giulia Tozzi, Lorena Travaglini, Alessandra Torraco, Anna Pastore, Marco Sparaco, Sara Petrillo, Rosalba Carrozzo, Enrico Bertini, Fiorella Piemonte

**Affiliations:** 1 Unit for Neuromuscular and Neurodegenerative Diseases, Children’s Hospital and Research Institute “Bambino Gesù”, Piazza S. Onofrio 4, 00165 Rome, Italy; E-Mails: carletti.barbara@tiscali.it (B.C.); emanuela.piermarini@opbg.net (E.P.); giulia.tozzi@opbg.net (G.T.); lorena.travaglini@opbg.net (L.T.); alessandra.torraco@opbg.net (A.T.); sara.petrillo@opbg.net (S.P.); rosalba.carrozzo@opbg.net (R.C.); enricosilvio.bertini@opbg.net (E.B.); 2 Biochemistry Laboratory, Children’s Hospital and Research Institute “Bambino Gesù”, Piazza S. Onofrio 4, 00165 Rome, Italy; E-Mail: anna.pastore@opbg.net; 3 Division of Neurology, Department of Neurosciences, Azienda Ospedaliera, “G. Rummo”, Via Pacevecchia 53, 82100 Benevento, Italy; E-Mail: marcosparaco@alice.it

**Keywords:** Friedreich’s ataxia, neurodegeneration, glutathione, oxidative stress, mitochondrial enzymes

## Abstract

Friedreich’s ataxia (FRDA) is a hereditary neurodegenerative disease characterized by a reduced synthesis of the mitochondrial iron chaperon protein frataxin as a result of a large GAA triplet-repeat expansion within the first intron of the frataxin gene. Despite neurodegeneration being the prominent feature of this pathology involving both the central and the peripheral nervous system, information on the impact of frataxin deficiency in neurons is scant. Here, we describe a neuronal model displaying some major biochemical and morphological features of FRDA. By silencing the mouse NSC34 motor neurons for the frataxin gene with shRNA lentiviral vectors, we generated two cell lines with 40% and 70% residual amounts of frataxin, respectively. Frataxin-deficient cells showed a specific inhibition of mitochondrial Complex I (CI) activity already at 70% residual frataxin levels, whereas the glutathione imbalance progressively increased after silencing. These biochemical defects were associated with the inhibition of cell proliferation and morphological changes at the axonal compartment, both depending on the frataxin amount. Interestingly, at 70% residual frataxin levels, the *in vivo* treatment with the reduced glutathione revealed a partial rescue of cell proliferation. Thus, NSC34 frataxin silenced cells could be a suitable model to study the effect of frataxin deficiency in neurons and highlight glutathione as a potential beneficial therapeutic target for FRDA.

## 1. Introduction

Frataxin is a ubiquitous mitochondrial iron-binding protein involved in the biosynthesis of Fe/S clusters and heme. A defect of this protein causes FRDA, a severe neurodegenerative disease affecting the upper motor neurons of the corticospinal tract, the posterior columns of the spinal cord, the spinocerebellar tract and the large sensory fibres of the peripheral nerves. Although the role of frataxin in mammalian cells is still controversial, it is widely accepted that this protein is involved in mitochondrial iron homeostasis, biosynthesis of the Fe/S complexes (ISC), stimulation of the oxidative phosphorylation and regulation of the oxidative stress [1–5]. Frataxin deficiency has been investigated in several cell lines such as patient-specific immortalized lymphoblasts, primary fibroblasts, peripheral lymphocytes, or HeLa cells [6–10], as well as established cell lines in which frataxin levels were reduced by antisense gene expression [11] or RNA interference [12–16]. Furthermore, several induced pluripotent stem cell (iPSC) lines have been generated using different combinations of reprogramming transcription factors [17–19]. However, to date, little information is available on the selective vulnerability of specific neurons to frataxin deficiency and a good model in neuronal cells to reproduce the pathophysiology of FRDA is still lacking.

In a previous paper, we silenced murine NSC34 neuroblastoma cells, deriving from the fusion with spinal cord motor neurons, for the gene of frataxin and evaluated the expression of the transcription factor Nrf2 that has a fundamental role in the antioxidant response to oxidative stress [20]. We found a decrease of Nrf2 expression in NSC34 as a consequence of frataxin deficiency [21]. This loss of Nrf2 may greatly enhance the neuronal susceptibility to oxidative stress and make FRDA neurons particularly vulnerable to oxidative injury. Thus, in order to tentatively define a correlation between frataxin silencing and the severity of the neuronal degeneration, in this study we have developed two cell lines of frataxin-silenced NSC34 motor neurons expressing 70% and 40% residual amounts of frataxin, and we have analyzed the mitochondrial function, the glutathione homeostasis and the axonal morphology in neurons carrying different percentages of frataxin silencing.

Evidence of oxidative damage has been long demonstrated in FRDA and proposed as the origin of the disease [3,22,23]. Various degrees of mitochondrial impairment and oxidative stress have been shown in patient’s derived tissues or in cell and animal models [3,24–29]. Increased levels of oxidative stress markers (plasma malondialdehyde, urine 8-hydroxy-2-deoxyguanosine) have been found in patients with FRDA [30,31], and improvement of cardiac and skeletal muscle bioenergetics have been observed after antioxidant treatment [32]. A decreased glutathione (GSH) concentration and a significant elevation of glutathionyl-hemoglobin have also been reported in blood and fibroblasts of patients with FRDA [28,29]. Nevertheless, the role of oxidative stress in FRDA is still a matter of debate. No evidence has been found in conditional knockout mouse models [12], whereas “humanized” GAA repeat expansion mouse models of FRDA showed clear signs of oxidative stress leading to progressive neuronal and cardiac pathology [13,33]. Thus, the pathogenic mechanism causing neurodegeneration in FRDA remains currently unknown and no treatments have been proven to delay, prevent, or reverse the neurodegenerative progression. The 70% and 40% frataxin-silenced NSC34 motor neurons reproduce several major biochemical and morphological features related to FRDA and may represent a useful model to evaluate the effect of frataxin deficiency on neurodegeneration.

## 2. Results and Discussion

### 2.1. Frataxin Silencing in NSC34 Motor Neurons

**Figure 1.**
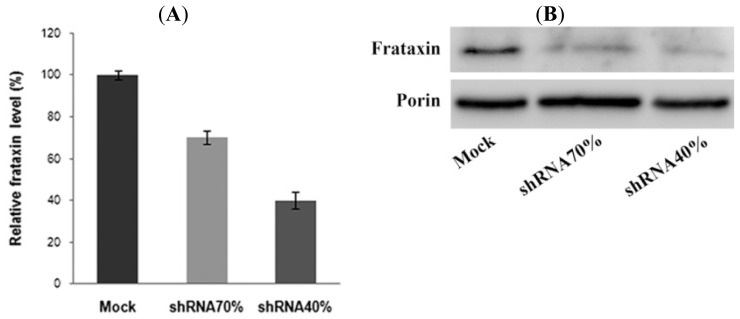
Efficiency of the frataxin shRNA. Proliferating NSC34 cells were transduced with lentivectors encoding for shRNA sequences against murine frataxin (shRNA 70% and shRNA 40%) or containing only the GFP reporter gene and a shRNA non silencing insert (mock control). (**A**) The level of frataxin mRNA was quantified by RT-PCR. The results were expressed as percentage of the residual amounts of frataxin in respect to control mock-transfected cells; (**B**) Representative western blot of the frataxin protein level. Porin was used as the reference protein.

Frataxin gene silencing has been carried out by means of specific shRNA lentiviral vectors. By real time-PCR (RT-PCR) (Figure 1A), we obtained a 30%–60% decrease of the frataxin mRNA, compared to the level of frataxin mRNA in the control cell line consisting of cells infected with the GFP non-silencing vector (reported as Mock). Specifically, two cell lines (indicated as shRNA 70% and shRNA 40%) have been obtained, with 70% and 40% residual amounts of frataxin, respectively (Figure 1B).

### 2.2. Frataxin-Silenced NSC34 Cells Show a Decrease of Cell Proliferation Rate

**Figure 2.**
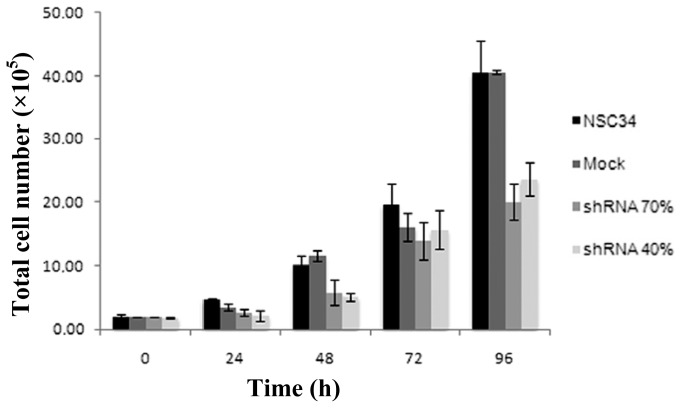
Viability of the stably frataxin-silenced NSC34 cells. Cell proliferation was evaluated by trypan blue exclusion assay. The data were expressed as number of cells that excluded the dye. Mean of three experiments in quadruplicate.

To quantify cell proliferation after frataxin silencing, we analyzed the number of viable cells at 24, 48, 72, 96 h of growth. As reported in Figure 2, frataxin deficiency slows down cell proliferation, particularly on the fourth day, when the total viable cells were about 49% in the shRNA 70% and 58% in the shRNA 40% lines, with respect to the control mock line. Thus, in our model a 70% of frataxin residual amount is sufficient to determine a proliferation defect and may be useful for screening the effect of a library of new therapeutic drugs.

### 2.3. Frataxin Deficiency Specifically Affects the Mitochondrial Complex I

**Figure 3.**
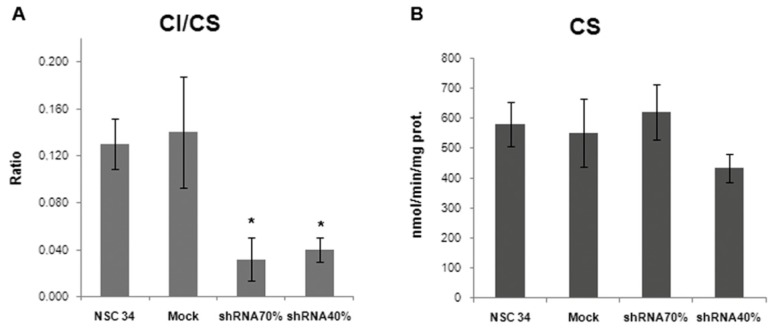
(**A**) Complex I activity was measured on isolated mitochondria as reported in Experimental Section and expressed as ratio of the CS activity; (**B**) The CS activity was assayed in isolated mitochondria and expressed as nmol/min/mg of proteins. Mean and SD of five experiments (^*^ *p* < 0.05).

**Figure 4.**
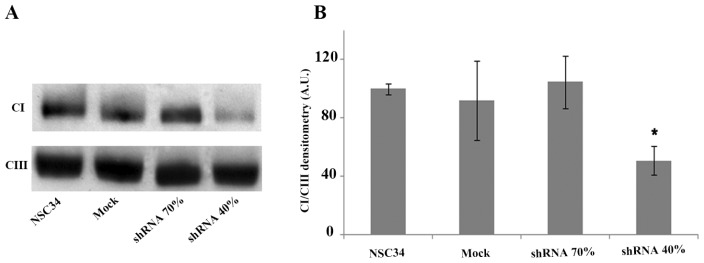
(**A**) Western blotting of BNGE performed on mitochondria isolated from NSC34, Mock, shRNA 70% and shRNA 40%. shRNA 40% cells show a reduction of CI of about 45% when compared to the NSC34 and Mock cells. For CI and CIII visualization, NDUFA9 and Core2 antibodies were used respectively, as reported in Experimental Section; (**B**) Densitometry analysis of the BNGE showing CI normalized to CIII amounts. The results are the mean of three different experiments (^*^ *p* < 0.05).

Numerous studies using patient’s derived tissues or various cell and animal models have established that frataxin deficiency results in varying degrees of respiratory chain impairment. Thus, we decided to isolate mitochondria from silenced NSC34 and to measure the enzymatic activities of the mitochondrial respiratory chain enzymes in shRNA 70% and shRNA 40% NSC34 cells (Table 1). Complex I, III and IV showed decreased activities in frataxin-deficient neurons but, interestingly, only Complex I (CI) reached the statistical significance (*p* < 0.05), with a CI/Citrate synthase (CS) ratio of 23% in shRNA 70% and 28% in shRNA 40%, compared to the control mock cells (Figure 3A). CS activity, an index of mitochondrial content, did not show any significant differences among controls and both silenced NSC34 cell lines (Figure 3B), thus indicating a specific inhibition of CI, not involving the number of mitochondria. These findings suggest the presence of a specific frataxin threshold in motoneuronal cells, where a 70% residual frataxin amount is already pathological for motor neurons, causing complex I impairment at the same extent of the 40%. To assess whether the reduction of CI activity was paralleled by a decrease in the amount of the fully assembled enzyme, we performed the BNGE followed by western blotting using specific antibodies directed against respiratory chain complexes subunits. As shown in Figure 4, mitochondria isolated from shRNA 40% showed an approximately 45% reduction of CI, when compared to the mock control. No lower molecular weight complexes were evident in the shRNA40%, indicating that a reduced level of frataxin affected the stability of CI more than its assembly. Complex III had similar amounts in all samples and was used as internal loading control. Notably, despite the significant inhibition of activity, CI expression was not reduced in the shRNA 70% by BNGE (Figure 4), thus indicating an early down-regulation of the enzyme activity in silenced motor neurons, preluding the loss of protein expression.

### 2.4. Frataxin Deficiency Alters the Glutathione Homeostasis in NSC34 Cells

**Figure 5.**
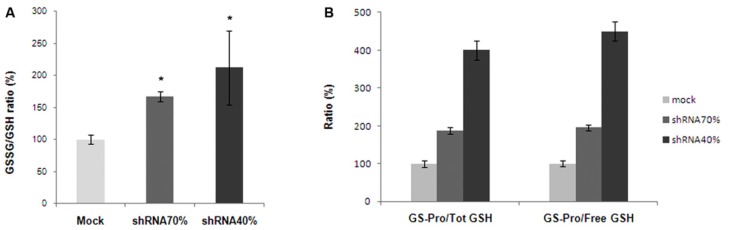
HPLC analysis of glutathione forms in frataxin-silenced NSC34 cells. GSSG/GSH ratio (**A**); and protein-bound (GS-Pro)/total (Tot) GSH and protein-bound/free GSH ratios (**B**) were determined in NSC34, Mock, shRNA 70% and shRNA 40% cells. ^*^ *p* < 0.05.

Glutathione is essential for neuronal detoxification of reactive oxygen species (ROS) and hypersensitivity to oxidants has been evidenced in several cellular and animals models of FRDA [8,27,34–36]. Thus, glutathione homeostasis has been analyzed in the shRNA 70% and 40% silenced cells, in the control mock and in NSC34 cell line, and the balance between GSSG and GSH (Figure 5A) and between GS-Pro/Tot GSH and GS-Pro/Free GSH ratios (Figure 5B) were determined. The GSSG/GSH ratios increased of about 1.7 in shRNA 70% and two folds in 40% frataxin-silenced NSC34 cells, with respect to the mock control. Also the GS-Pro/Tot GSH and GS-Pro/Free GSH ratios progressively increased after silencing, thus supporting an oxidative imbalance in both frataxin deficient neurons. This shift of the cellular redox equilibrium toward more oxidized forms of glutathione interestingly parallels the trend of oxidation reported in fibroblasts and in blood cells of patients with FRDA [28,29].

### 2.5. Frataxin Silencing Affects the Neuronal Morphology of NSC34 Cells

**Figure 6.**
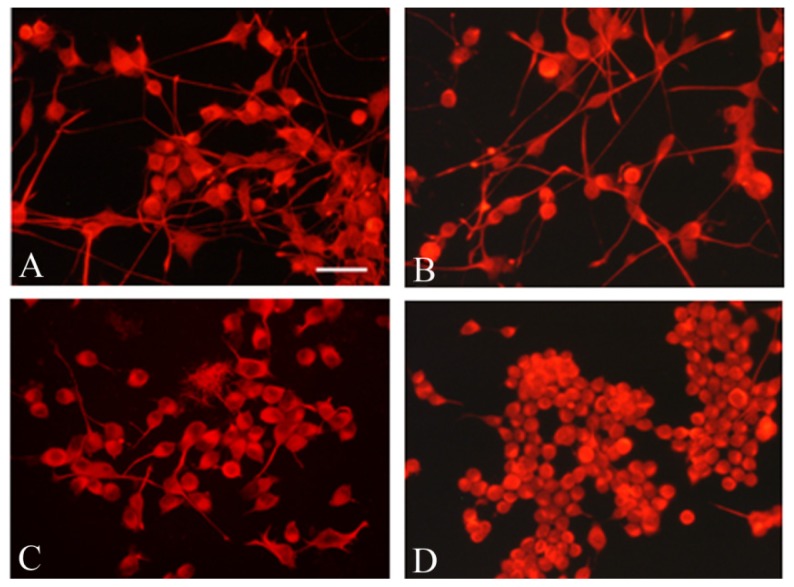
Morphology of NSC34 (**A**); Mock (**B**); shRNA 70% (**C**) and shRNA 40% (**D**) induced to differentiate for six d.i.v. The neuronal soma and axonal processes were immunostained with an antibody against the neurofilament heavy chain subunit (NF-H). Bar = 50 μm.

The prominent neurodegenerative feature in FRDA is a distal length-related axonal degeneration. Thus, in order to study whether frataxin gene silencing alters the NSC34 cell morphology, we analyzed their neuronal differentiation following frataxin deficiency. The immunocytochemistry demonstrated that the differentiation marker Neurofilament Heavy chain (NF-H) was highly expressed in the frataxin-deficient neuron-like cells (Figure 6). However, the morphometric analysis showed that shRNA 40% silenced cells (Figure 6D) were almost devoid of neurites, when compared to mock (Figure 6B) and to NSC34 cells (Figure 6A), whereas the shRNA 70% neurons (Figure 6C) still displayed short processes. This pattern resembles the morphology recently reported for NSC34 after *in vitro* treatment with an oxidant stimulus [37], and suggests a role for frataxin deficiency in the axonal retraction and neurodegeneration.

### 2.6. The in Vitro GSH-Treatment Restores the Proliferation Rate of Silenced NSC34 Cells

**Figure 7.**
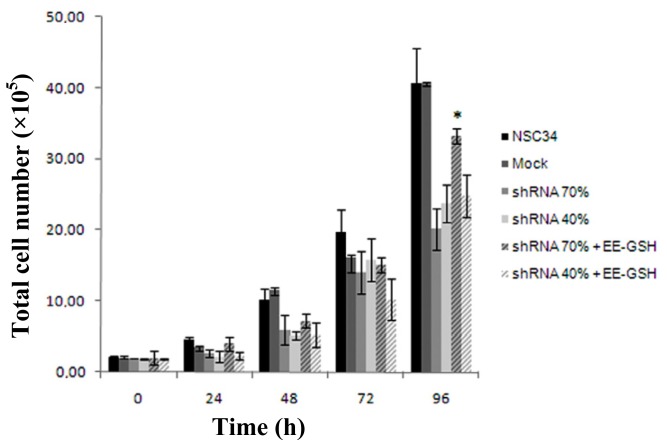
Effect of EE-GSH on cell proliferation of frataxin-silenced NSC34. After incubating cells with 500 μM EE-GSH, cell proliferation was evaluated by trypan blue exclusion assay. The data were expressed as the number of cells that excluded the dye. Results represent the mean of three independent determinations repeated four times. ^*^ *p* < 0.05.

As frataxin deficiency leads to a slowdown of the cell proliferation and the GSSG might be the mediator of this growth decline, we treated both shRNA 70% and 40% silenced NSC34 cells with EE-GSH, a membrane-permeable form of the reduced glutathione, in order to evaluate if restoring the correct GSSG/GSH ratio may affect cell proliferation. Interestingly, after 4 days of growth, EE-GSH induced a 1.6-fold increase of shRNA 70% cell proliferation, with respect to untreated shRNA 70%, and this almost equated the control mock growth rates (Figure 7). In contrast, no recovery was observed when shRNA 40% neurons were incubated in the presence of EE-GSH, indicating that 60% frataxin silencing may trigger a cascade of harmful effects difficult to reverse. These findings evidence the importance of a correct intracellular ratio between oxidized and reduced glutathione and further support the existence of a pathogenic threshold in motor neurons.

### 2.7. Discussion

The pathogenic mechanism causing neurodegeneration in FRDA is still uncertain. So far, few neuronal cell models have been developed for this disease and only few data are available on the effect of frataxin deficiency in neurons.

Here, we describe a neuronal cell model of frataxin deficiency obtained by silencing frataxin in motoneuronal cells, a neuroblastoma spinal cord hybrid cell line displaying motor neuron-like phenotype. To generate the neuronal cell model for frataxin deficiency, we used lentivirus carrying a shRNA construct. This approach led to a 30%–60% decrease of the frataxin mRNA and determined several biochemical and morphological modifications that are highly suggestive of some aspects of the axonal pathological profile of FRDA.

The stable frataxin silencing caused: (1) a significant reduction of the mitochondrial CI activity; (2) an imbalance of the main antioxidant GSH, with a consistent increase of its oxidized form GSSG; (3) the inhibition of the cell proliferation; and (4) the axonal degeneration.

Mitochondrial respiratory chain enzymes deficiencies are a distinct hallmark of FRDA. Impairment of complexes I/II/III and aconitase have been demonstrated in cardiac muscle samples from patients with FRDA [24,38], in lymphocytes [39] and in mouse models [25]. Defects in complex I and aconitase have also been observed in the cerebellum and dorsal root ganglia from patients’ tissues [38]. Accordingly, we found a significant reduction of mitochondrial complex I in frataxin-silenced NSC34 motor neurons, involving the protein in its fully assembled structure. Since complex I contains Fe/S cluster subunits, this result supports the hypothesis of a frataxin involvement in Fe/S cluster biosynthesis. Noteworthy, a 70% residual amount of frataxin was already pathological for motor neurons, causing complex I impairment at the same extent of the 40% frataxin level. This allows hypothesizing the presence of a cell threshold of pathogenicity in mouse motor neurons, at least with respect to the pathological feature analyzed in this study. Our finding is also consistent with a recent article of Hick *et al.* [19] showing that neurons from FRDA iPSCs develop a distinctive phenotype suggestive of mitochondrial respiratory chain defect, probably as a consequence of Fe/S enzyme deficiency or oxidative stress. A second important pathological feature emerging from our frataxin silenced model is the dramatic alteration of the GSSG/GSH balance, with a significant rise of all oxidized forms of glutathione, including the protein-bound one. These findings confirm the impairment of glutathione homeostasis previously described in fibroblasts and blood of FRDA patients [28,29] and support the role of glutathione as a potent redox indicator for this disease. In addition, since mitochondrial respiratory chain enzymes, especially complex I, generate significant levels of ROS even under physiological conditions and, at the same time, are sensitive targets of oxidative damage [40,41], a mitochondrial respiratory chain dysfunction may consistently contribute to ROS over-load and trigger a vicious circle, where the glutathione imbalance inhibits mitochondrial function further exacerbating the condition of oxidative stress. The oxidative stress can also be amplified by the loss of the transcription factor Nrf2, found to be depleted either in the NSC34 cell model [21] or in dorsal root ganglia and cerebella of a FRDA mouse model [33]. Since glutathione synthesis is regulated by Nrf2, the impairment of this pathway may greatly enhance the cellular susceptibility to oxidative stress and make FRDA neurons more vulnerable to injury.

The high sensitivity of FRDA neurons to oxidation affects cell proliferation. Indeed, both the shRNA 70% and 40% frataxin silenced cell lines exhibited a defect in cell proliferation, which was consistent with previous studies performed on HeLa cells [10] and murine fibroblasts [12]. To note, the *in vivo* treatment with the permeable form of GSH, able to establish the correct GSSG/GSH ratio, restored the cell proliferation rate suggesting that the increase of GSSG could be the basis of the neuronal damage and, very importantly, that this process might be reversible.

Neuronal cytoskeletal proteins are particularly sensitive to the oxidative stress and may represent a target of the axonal degeneration [37,42–44]. Indeed, when exposed to pro-oxidants, microtubules and microfilaments undergo marked alterations of their structure that also modify their ability to polymerize [45]. Therefore, the inability of our silenced motoneuronal cells to generate mature axonal processes may result from an altered intracellular redox environment affecting the polymerization of cytoskeletal proteins.

Our previous studies showing loss of the cytoskeletal organization in fibroblasts and spinal cord of patients with FRDA [28,46] and the constitutive glutathionylation of cytoskeletal proteins in human CNS [47] strengthen the hypothesis of glutathione as a redox-modulator of neuronal cytoskeleton assembly, axonal integrity and neuron vitality. The glutathione-mediated impairment of neuronal cytoskeleton could also contribute to explain the “dying-back” phenomenon characterizing the pattern of long tract degeneration in FRDA, although further studies are essential to elucidate this aspect of the pathology.

A critical aspect of the present study is that a 30% silencing of frataxin is sufficient to determine Complex I impairment, glutathione imbalance and proliferation defects in motor neurons. This may appear contrasting with evidence from human and mouse heterozygous carrier that are asymptomatic at ~40%–50% frataxin levels [48,49]. Nevertheless, recent reports demonstrated that similar to other haploinsufficiency diseases, the absence of clinical symptoms in FRDA heterozygous carriers does not necessarily mirror the absence of a biochemical phenotype [50–52]. The gene expression profile of peripheral blood mononuclear cells from FRDA patients compared to related heterozygous carriers and normal controls demonstrated that FRDA heterozygous (clinically asymptomatic) presented robust gene expression changes in line with the larger changes of the same genes observed in patients compared to normal controls [52]. These results are also consistent with similar data in animal models with no or mild clinical symptoms [50,51]. Thus, our work, according to the above mentioned articles suggests that a mild frataxin deficiency might reveal the presence of a biochemical phenotype which is informative of the pathological process observed in the disorder. In addition, our findings introduce, for the first time, the concept of a threshold of pathogenicity in FRDA and allow speculating that distinct thresholds of residual frataxin may induce differential responsiveness in different cell types. Interestingly, the cell proliferation rescue that we obtained after the GSH treatment occurs only when frataxin was at 70% residual levels indicating a threshold beyond which the process seems to become irreversible.

## 3. Experimental Section

### 3.1. Double-Stranded RNA Design

Lentiviral particle encoding short-hairpin RNA sequences were purchased from Open Biosystem (Waltham, MA, USA) and they contained the sequences against mouse frataxin hereafter reported as shRNA 70% and shRNA 40%. Control cell lines consisted of cells infected with the lentiviral vector containing the *GFP* gene and a shRNA non-silencing insert referred to as mock. The hairpin stem consists of 22 nt of dsRNA and a 19 nt loop from mouse miR30. Adding the miR30 loop and 125 nt of miR30 flanking sequence on either side of the hairpin results in greater than 10-fold increase in Drosha and Dicer processing of the expressed hairpins, when compared with conventional shRNA designs without microRNA [53]. Each silencing shRNA construct has been sequence verified before being cloned into the vector to ensure a match to the target gene.

### 3.2. Cell Culture

The NSC34 neuroblastoma cells, deriving from the fusion with spinal cord motor neurons (originally donated by Neil Cashman), were maintained in Dulbecco’s Modified Eagle Medium (DMEM, Invitrogen, Grand Island, NY, USA) supplemented with 10% fetal bovine serum (Invitrogen), 1% penicillin-streptomycin (20 U/mL, Invitrogen) and 1% glutamine (2 mM, Invitrogen). Cells were differentiated as described previously [37]. Briefly, cells were cultured at a density of 2000 cell/cm^2^ on tissue culture glass cover slips coated with 100 μg/mL of poly-d-lysine (Sigma, St. Louis, MO, USA). Cells were maintained for 6–7 days *in vitro* (d.i.v.) at 37 °C in a humidified atmosphere with 5% CO_2_ in DMEM/F12 (Invitrogen), supplemented with glutamine (2 mM; Invitrogen), penicillin-streptomycin (20 U/mL; Invitrogen), 1% fetal bovine serum (Invitrogen) and 1% non essential amminoacids (Sigma).

### 3.3. Stable shRNA Cell Lines Generation

For infections, NSC34 cells were plated overnight at a density of 2 × 10^4^. ShRNA lentiviral particle was diluted in maintaining medium without serum and antibiotics, and added to the cells for 5 h. Then, a further addition of complete medium was made to the cultures, and cells were collected after 72 h. Cell sorting was performed 96 h after infection. Trypsinized cells were resuspended in PBS and filtered through a 50 μm sterile mesh (BD Biosciences, Franklin Lakes, NJ, USA) and sorted for GFP expression by using a FACSAria II cell sorter (BD Biosciences). Positive cells with the strongest GFP expression were collected and underwent puromycin selection (10 μg/mL). Antibiotic selection was maintained for 7 days to obtain a stable cell line, which was finally analyzed for frataxin silencing.

### 3.4. Real-Time Quantitative PCR

Total RNA was extracted with RNAzolB (Tel-Test Inc., Friendswood, TX, USA) according to the manufacturer’s instructions and quantified spectrophotometrically. cDNA synthesis was performed with 1 μg of DNase-treated RNA using ImProm-II reverse transcriptase (Promega, Madison, WI, USA) and oligodT as primer. Real-time PCR was carried out with PCR primers specific for frataxin mRNA (sense, 5′-GTGGAGATCTAGGAACCTATG-3′, and antisense 5′-TTAAGGCTTTAGTGAGCTCTG-3′) and a specific fluorogenic probe (5′-TCCAGTCATAACGCTTAGGTCCAC-3′). The sense and antisense primers were designed to be complementary to regions on two different exons and the probe to span intron–exon boundaries to avoid amplification and recognition of genomic DNA. The mRNA encoding for the housekeeping gene glyceradehyde-3-phosphate dehydrogenase (GAPDH) was used as an endogenous reference. The PCR reaction was carried out using the GeneAmp™ 5700 Sequence Detection System (Applied Biosystems, Foster City, CA, USA). Results were normalized to GAPDH levels using the 2^−ΔΔ^*^C^*^t^ method. A value of 1 was given to control samples chosen as calibrators and the sample values expressed the *n*-fold reduction of frataxin mRNA with respect to the calibrator (normalized dose = nd).

### 3.5. Western Blot Analysis of Frataxin Levels

Cell pellets were homogenized with lysis buffer (Cell Signaling, Danvers, MA, USA) containing complete protease inhibitors and PSMF (Roche Applied Science, Penzberg, Germany). Forty micro grams of lysates were analyzed on 4%–12% Bis–Tris gels (Invitrogen). Electrophoresis was carried out according to the manufacturer’s recommendations. After electrophoresis, proteins were transferred to nitrocellulose membranes by the iBlot device (Invitrogen), blocked with Odyssey blocking buffer (LI-COR Biotechnology, Lincoln, NE, USA) for 1 h, and incubated overnight with the primary anti-Frataxin antibody (1:250, Millipore, Temecula, CA, USA). An antibody against porin (Mitosciences, Abcam, UK) was used as the reference protein (1:10,000 dilution).

### 3.6. Cell Proliferation Assay

Cell proliferation was assessed by trypan blue exclusion assay. Briefly, parental and stably transfected cell lines were seeded in T25 flasks (2 × 10^5^ cells per flask) and cultured for 5 days. At 24, 48, 72 and 96 h, cells were trypsinized and the number of viable cells was visually counted with a hemocytometer. Four counts were performed per flask on three different experiments. The treatment with Ethyl-Ester-Glutathione (EE-GSH) has been performed by incubating cells with 500 μM of the compound (final concentration) for the period of the culture.

### 3.7. Immunocytochemistry

Immunostaining was performed on cells cultured on coverslips and fixed for 10 min at 4 °C with 0.12 M phosphate buffer containing 4% paraformaldehyde. After several washes, cells were permeabilized with PBS containing 1.5% normal serum and 0.25% Triton X-100. Cells were then incubated for 2 h, at room temperature, with a neuronal specific anti-rabbit polyclonal antibody directed against the neurofilament heavy chain subunit (NF-H, 1:100, Sigma, St. Louis, MO, USA). Subsequently, cells underwent three washes for about 10 min each and then incubated for 1 h at room temperature with the secondary antibody conjugated with alexa fluor 555 (1:500, Molecular Probes, Eugene, OR, USA). Glass cover slips were mounted on microscope slides with Tris–glycerol supplemented with 10% Mowiol (Calbiochem, La Jolla, CA, USA) to reduce fading of fluorescence.

### 3.8. Mitochondria Isolation

NSC34 cells (6 × 10^6^), pelleted by centrifugation at 300× *g* (5 min, at room temperature), were suspended in lysis buffer (1 mL) containing 250 mM saccharose, 1 mM EGTA, 0.5% BSA, 10 mM HEPES (pH 7.4) and centrifuged at 4 °C for 8 min at 1089× *g*. The supernatant was collected and centrifuged at 4 °C for 12 min at 14,600× *g*. The pellet was resuspended in 100 μL of suspension buffer (250 mM saccharose, 10 mM Tris, 1 mM EDTA, pH 7.4) and the mitochondria obtained were immediately used for enzyme activity assays.

### 3.9. Determination of Mitochondrial Respiratory Chain Enzyme Activities

Isolated mitochondria were resuspended in wash buffer containing 250 mM saccharose, 1 mM EDTA, 10 mM Tris (pH 7.4) and all enzyme activities were measured as we previously described [54]. Briefly, Complex I (NADH:CoQ oxidoreductase, EC 1.6.5.3) activity was measured by recording the decrease in absorbance due to oxidation of NADH at 340 nm (ɛ = 6.81 mM^−1^·cm^−1^). Specificity of Complex I activity was measured by the percent inhibition after addition of the Complex I inhibitor rotenone (10 μM). Complex II (succinate:Co Q oxidoreductase, EC 1.3.5.1) was determined by following the reduction of 2,6-dichlorophenol indophenol coupled with reduction of decylubiquinone (DB) at 600 nm (ɛ = 19.1 mM^−1^·cm^−1^). The reaction was started by the addition of 16 mM succinate and 50 μM DB. Complex III (CoQ:cytocrome c oxidoreductase, EC 1.10.2.2) activity was assayed by following the reduction of cytocrome c at 550 nm (ɛ = 18.5 mM^−1^·cm^−1^). The assay was started by the addition of 50 μM DBH2. Complex IV (cytochrome c oxidase, EC 1.9.3.1) activity was measured by following the oxidation of reduced cytochrome c at 550 nm (ɛ = 18.5 mM^−1^·cm^−1^). Reduced cytochrome c was prepared immediately before use by adding a few grains of sodium dithionite. Citrate synthase (EC 2.3.3.1) activity was used as mitochondrial matrix enzymatic marker. The reaction was started by the addition of 0.4 mM oxalacetate and the initial rate was measured following the reduction of DTNB at 412 nm (ɛ = 13.6 mM^−1^·cm^−1^). Protein concentrations were quantified by BCA-protein assay (Sigma-Aldrich, St. Louis, MO, USA).

### 3.10. HPLC Determination of Various Forms of Glutathione

Measurements of the reduced (GSH), oxidized (GSSG) and protein-bound (GS-Pro) glutathione were performed as we previously described [28]. Cells were sonicated three times for 2 s in 0.1 mL of 0.1 M potassium phosphate buffer (pH 7.2). After sonication, 50 μL of 12% sulfosalicylic acid were added, and the GSH content in the acid-soluble fraction was determined (free GSH). The protein pellet was dissolved in 150 μL of 0.1 N NaOH, and GS-Pro was determined. To measure GSSG, cells were sonicated three times for 2 s in 0.1 mL of 0.1 M potassium phosphate buffer (pH 7.2) containing 10 mM *N*-ethylmaleimide. Total GSH was determined in cell lysates before adding 12% sulfosalicylic acid. GSH levels were calculated by subtracting GSSG concentrations from free GSH values. Derivatization and chromatography procedures were carried out as we previously reported [55].

### 3.11. Blue Native Gel Electrophoresis (BNGE)

Native respiratory chain complexes were separated by BNGE according to the previous reported protocol [56]. Briefly, 40 μg of mitochondrial proteins were loaded on a linear 5%–13% gradient non-denaturating gel. Proteins were subsequently transferred to a PVDF membrane and blotted with specific antibodies directed against CI and CIII subunits (NDUFA9 and Core2, respectively). Cross-reacting material was visualized using Chemiluminescent HRP Substrate detection kit (Millipore Corporation, Billerica, MA, USA). Densitometry analysis was performed using the Quantity One Software (BioRad, Hercules, CA, USA).

### 3.12. Statistical Analysis

Data are expressed as mean ± SD. The comparison between values obtained in treated samples and controls was performed by the Student *t*-test for unpaired data.

## 4. Conclusions

Overall, our silenced neuronal cell line represents a suitable model to elucidate the mechanism by which frataxin deficiency causes neuronal damage in FRDA. In recent years, the ability to generate disease-specific iPSC lines from patients and to differentiate them into target neurons provided a powerful tool for the study of FRDA and several groups showed the successful differentiation of iPSC peripheral sensory neurons from patients’ derived fibroblasts [18,19,57,58]. These new model systems as well as neural stem cells from humanized FRDA mice [58] are very promising to recapitulate at least in part the neurodegenerative process. Nonetheless, the heterogeneity of the cultures and the difficulties to generate large numbers of severely affected neurons (*i.e.*, large proprioceptive sensory neurons) still represent a limitation of these new methods. We think that the present model might provide a useful and synergistic strategy both for investigating the FRDA neurodegenerative process and for screening new therapeutic molecules.

We showed that a defect in mitochondrial complex I activity, caused by an incorrect assembly of the protein, is accompanied by morphological changes at the axon compartment and by an inhibition of the cell proliferation. According to previous studies, the impairment of the activity of iron sulfur proteins was associated with oxidative stress, with a consistent increase of the GSSG level in silenced neurons. In addition, our findings suggest the existence of a pathological threshold responding to the beneficial effect of antioxidant treatments and this may help to identify specific tissue thresholds critical for evaluating the efficacy of new therapeutic compounds.

## Figures and Tables

**Table 1.** Effect of frataxin silencing on mitochondrial electron transport complex activities.

Enzyme	NSC34	Mock	shRNA 70%	shRNA 40%
CI	0.13 ± 0.02	0.16 ± 0.05	0.03 ± 0.02	0.04 ± 0.01 *
CII	0.08 ± 0.01	0.07 ± 0.02	0.06 ± 0.02	0.08 ± 0.02
CIII	0.21 ± 0.09	0.23 ± 0.07	0.18 ± 0.03	0.12 ± 0.03
CIV	1.97 ± 0.32	2.11 ± 0.36	1.83 ± 0.31	1.20 ± 0.20
CS	579 ± 73.71	550 ± 114.30	620 ± 91.40	434 ± 47.32

Values are expressed as nmol/min/mg of mitochondrial protein and represent means (*n* = 5) ± SD.

* *p* < 0.05.

## Abbreviations

FRDA: Friedreich’s Ataxia
Nrf2: NF-E2-related factor
GSH: reduced glutathione
GSSG: oxidized glutathione
CNS: central nervous system
BNGE: blue native gel electrophoresis

## References

[1] Martelli A., Wattenhofer-Donzé M., Schmucker S., Bouvet S., Reutenauer L., Puccio H. (2007). Frataxin is essential for extramitochondrial Fe–S cluster proteins in mammalian tissues. Hum. Mol. Genet.

[2] Richardson D.R., Huang M.L., Whitnall M., Becker E.M., Ponka P., Suryo Rahmanto Y. (2010). The ins and outs of mitochondrial iron-loading: The metabolic defect in Friedreich’s ataxia. J. Mol. Med.

[3] Santos R., Lefevre S., Sliwa D., Seguin A., Camadro J.M., Lesuisse E. (2010). Friedreich ataxia: Molecular mechanisms, redox considerations, and therapeutic opportunities. Antioxid. Redox Signal.

[4] Schmucker S., Martelli A., Colin F., Page A., Wattenhofer-Donzé M., Reutenauer L., Puccio H. (2011). Mammalian frataxin: an essential function for cellular viability through an interaction with a preformed ISCU/NFS1/ISD11 iron-sulfur assembly complex. PLoS One.

[5] Shan Y., Cortopassi G. (2012). HSC20 interacts with frataxin and is involved in iron-sulfur cluster biogenesis and iron homeostasis. Hum. Mol. Genet.

[6] Jauslin M.L., Wirth T., Meier T., Schoumacher F.A. (2002). Cellular model for Friedreich ataxia reveals small-molecule glutathione peroxidase mimetics as novel treatment strategy. Hum. Mol. Genet.

[7] Sturm B., Bistrich U., Schranzhofer M., Sarsero J.P., Rauen U., Scheiber-Mojdehkar B., de Groot H., Ioannou P., Petrat F. (2005). Friedreich’s ataxia, no changes in mitochondrial labile iron in human lymphoblasts and fibroblasts: A decrease in antioxidative capacity?. J. Biol. Chem.

[8] Wong A., Yang J., Cavadini P., Gellera C., Lonnerdal B., Taroni F., Cortopassi G. (1999). The Friedreich’s ataxia mutation confers cellular sensitivity to oxidant stress which is rescued by chelators of iron and calcium and inhibitors of apoptosis. Hum. Mol. Genet.

[9] Haugen A.C., di Prospero N.A., Parker J.S., Fannin R.D., Chou J., Meyer J.N., Halweg C., Collins J.B., Durr A., Fischbeck K. (2010). Altered gene expression and DNA damage in peripheral blood cells from Friedreich’s ataxia patients: Cellular model of pathology. PLoS Genet.

[10] Zanella I., Derosas M., Corrado M., Cocco E., Cavadini P., Biasiotto G., Poli M., Verardi R., Arosio P. (2008). The effects of frataxin silencing in HeLa cells are rescued by the expression of human mitochondrial ferritin. Biochim. Biophys. Acta.

[11] Santos M.M., Ohshima K., Pandolfo M. (2001). Frataxin deficiency enhances apoptosis in cells differentiating into neuroectoderm. Hum. Mol. Genet.

[12] Calmels N., Schmucker S., Wattenhofer-Donzé M., Martelli A., Vaucamps N., Reutenauer L., Messaddeq N., Bouton C., Koenig M., Puccio H. (2009). The first cellular models based on frataxin missense mutations that reproduce spontaneously the defects associated with Friedreich ataxia. PLoS One.

[13] Lu C., Schoenfeld R., Shan Y., Tsai H.J., Hammock B., Cortopassi G. (2009). Frataxin deficiency induces Schwann cell inflammation and death. Biochim. Biophys. Acta.

[14] Napoli E., Morin D., Bernhardt R., Buckpitt A., Cortopassi G. (2007). Hemin rescues adrenodoxin, heme a and cytochrome oxidase activity in frataxin-deficient oligodendroglioma cells. Biochim. Biophys. Acta.

[15] Palomo G.M., Cerrato T., Gargini R., Diaz-Nido J. (2011). Silencing of *frataxin* gene expression triggers p53-dependent apoptosis in human neuron-like cells. Hum. Mol. Genet.

[16] Soragni E., Herman D., Dent S.Y., Gottesfeld J.M., Wells R.D., Napierala M. (2008). Long intronic GAA^*^TTC repeats induce epigenetic changes and reporter gene silencing in a molecular model of Friedreich ataxia. Nucleic Acids Res.

[17] Ku S., Soragni E., Campau E., Thomas E.A., Altun G., Laurent L.C., Loring J.F., Napierala M., Gottesfeld J.M. (2010). Friedreich’s ataxia induced pluripotent stem cells model intergenerational GAA^*^TTC triplet repeat instability. Cell Stem Cell.

[18] Liu J., Verma P.J., Evans-Galea M.V., Delatycki M.B., Michalska A., Leung J., Crombie D., Sarsero J.P., Williamson R., Dottori M. (2011). Generation of induced pluripotent stem cell lines from Friedreich ataxia patients. Stem Cell Rev.

[19] Hick A., Wattenhofer-Donzé M., Chintawar S., Tropel P., Simard J.P., Vaucamps N., Gall D., Lambot L., André C., Reutenauer L. (2013). Neurons and cardiomyocytes derived from induced pluripotent stem cells as a model for mitochondrial defects in Friedreich's ataxia. Dis. Model. Mech.

[20] Calkins M.J., Johnson D.A., Townsend J.A., Vargas M.R., Dowell J.A., Williamson T.P., Kraftm A.D., Lee J.M., Li J., Johnson J.A. (2009). The Nrf2/ARE pathway as a potential therapeutic target in neurodegenerative disease. Antioxid. Redox Signal.

[21] D’Oria V., Petrini S., Travaglini L., Priori C., Piermarini E., Petrillo S., Carletti B., Bertini E., Piemonte F. (2013). Frataxin deficiency leads to reduced expression and impaired translocation of NF-E2-related factor (Nrf2) in cultured motor neurons. Int. J. Mol. Sci.

[22] Al-Mahdawi S., Pinto R.M., Varshney D., Lawrence L., Lowrie M.B., Hughes S., Webster Z., Blake J., Cooper J.M., King R. (2006). GAA repeat expansion mutation mouse models of Friedreich ataxia exhibit oxidative stress leading to progressive neuronal and cardiac pathology. Genomics.

[23] Armstrong J.S., Khdour O., Hecht S.M. (2010). Does oxidative stress contribute to the pathology of Friedreich’s ataxia? A radical question. FASEB J.

[24] Rotig A., de Lonlay P., Chretien D., Foury F., Koenig M., Sidi D., Munnich A., Rustin P. (1997). Aconitase and mitochondrial Fe/S protein deficiency in Friedreich ataxia. Nat. Genet.

[25] Puccio H., Simon D., Cossee M., Criqui-Filipe P., Tiziano F., Melki J., Hindelang C., Matyas R., Rustin P., Koenig M. (2001). Mouse models for Friedreich ataxia exhibit intramitochondrial cardiomyopathy, sensory nerve defect and Fe–S enzyme deficiency followed by iron deposits. Nat. Genet.

[26] Auchère F., Santos R., Planamente S., Lesuisse E., Camadro J.M. (2008). Glutathione-dependent redox status of frataxin-deficient cells in a yeast model of Friedreich’s ataxia. Hum. Mol. Genet.

[27] Bulteau A.L., Dancis A., Gareil M., Montagne J.J., Camadro J.M., Lesuisse E. (2007). Oxidative stress and protease dysfunction in the yeast model of Friedreich ataxia. Free Radic. Biol. Med.

[28] Pastore A., Tozzi G., Gaeta L.M., Bertini E., Serafini V., di Cesare S., Bonetto V., Casoni F., Carrozzo R., Federici G. (2003). Actin glutathionylation increases in fibroblasts of patients with Friedreich’s ataxia: A potential role in the pathogenesis of the disease. J. Biol. Chem.

[29] Piemonte F., Pastore A., Tozzi G., Tagliacozzi D., Santorelli F.M., Carrozzo R., Casali C., Damiano M., Federici G., Bertini E. (2001). Glutathione in blood of patients with Friedreich’s ataxia. Eur. J. Clin. Investig.

[30] Edmond M., Lepage G., Vanasse M., Pandolfo M. (2000). Increased levels of plasma malondialdehyde in Friedreich ataxia. Neurology.

[31] Schulz J.B., Dehmar T., Schols L., Mende H., Hardt C., Vorgerd M., Bürk K., Matson W., Dichgans J., Beal M.F. (2000). Oxidative stress in patients with Friedreich ataxia. Neurology.

[32] Schulz J.B., Boesch S., Bürk K., Dürr A., Giunti P., Mariotti C., Pousset F., Schöls L., Vankan P., Pandolfo M. (2009). Diagnosis and treatment of Friedreich ataxia: A European perspective. Nat. Rev. Neurol.

[33] Shan Y., Schoenfeld R.A., Hayashi G., Napoli E., Akiyama T., Iodi Carstens M., Carstens E.E., Pook M.A., Cortopassi G.A. (2013). Frataxin deficiency leads to defects in expression of antioxidants and Nrf2 expression in dorsal root ganglia of the Friedreich’s ataxia YG8R mouse model. Antioxid. Redox Signal.

[34] Calabrese V., Lodi R., Tonon C., D’Agata V., Sapienza M., Scapagnini G., Mangiameli A., Pennisi G., Stella A.M., Butterfield D.A. (2005). Oxidative stress, mitochondrial dysfunction and cellular stress response in Friedreich’s ataxia. J. Neurol. Sci.

[35] Lodi R., Tonon C., Calabrese V., Schapira A.H. (2006). Friedreich’s ataxia: From disease mechanisms to therapeutic interventions. Antioxid. Redox Signal.

[36] Llorens J.V., Navarro J.A., Martínez-Sebastián M.J., Baylies M.K., Schneuwly S., Botella J.A., Moltó M.D. (2007). Causative role of oxidative stress in a Drosophila model of Friedreich ataxia. FASEB J.

[37] Carletti B., Passarelli C., Sparaco M., Tozzi G., Pastore A., Bertini E., Piemonte F. (2011). Effect of protein glutathionylation on neuronal cytoskeleton: A potential link to neurodegeneration. Neuroscience.

[38] Bradley J.L., Blake J.C., Chamberlain S., Thomas P.K., Cooper J.M., Schapira A.H. (2000). Clinical, biochemical and molecular genetic correlations in Friedreich’s ataxia. Hum. Mol. Genet.

[39] Heidari M.M., Houshmand M., Hosseinkhani S., Nafissi S., Khatami M. (2009). Complex I and ATP content deficiency in lymphocytes from Friedreich’s ataxia. Can. J. Neurol. Sci.

[40] Taylor E.R., Hurrell F., Shannon R.J., Lin T.K., Hirst J., Murphy M.P. (2003). Reversible glutathionylation of Complex I increases mitochondrial superoxide formation. J. Biol. Chem.

[41] Papa S., de Rasmo D., Scacco S., Signorile A., Technikova-Dobrova Z., Palmisano G., Sardanelli A.M., Papa F., Panelli D., Scaringi R. (2008). Mammalian Complex I: A regulable and vulnerable pacemaker in mitochondrial respiratory function. Biochim. Biophys. Acta.

[42] Mieyal J.J., Chock P.B. (2012). Posttranslational modification of cysteine in redox signaling and oxidative stress: Focus on *S*-glutathionylation. Antioxid. Redox Signal.

[43] Sabens Liedhegner E.A., Gao X.H., Mieyal J.J. (2012). Mechanisms of altered redox regulation in neurodegenerative diseases-focus on *S*-glutathionylation. Antioxid. Redox Signal.

[44] Pastore A., Piemonte F. (2012). *S*-Glutathionylation signaling in cell biology: Progress and prospects. Eur. J. Pharm. Sci.

[45] Dalle-Donne I., Rossi R., Milzani A., di Simplicio P., Colombo R. (2001). The actin cytoskeleton responses to oxidants: From small heat shock protein phosphorylation to changes in the redox state of actin itself. Free Radic. Biol. Med.

[46] Sparaco M., Gaeta L.M., Santorelli F.M., Passarelli C., Tozzi G., Bertini E., Simonati A., Scaravilli F., Taroni F., Duyckaerts C. (2009). Friedreich’s ataxia: Oxidative stress and cytoskeletal abnormalities. J. Neurol. Sci.

[47] Sparaco M., Gaeta L.M., Tozzi G., Bertini E., Pastore A., Simonati A., Santorelli F.M., Piemonte F. (2006). Protein glutathionylation in human central nervous system: Potential role in redox regulation of neuronal defense against free radicals. J. Neurosci. Res.

[48] Pianese L., Turano M., lo Casale M.S., de Biase I., Giacchetti M., Monticelli A., Criscuolo C., Filla A., Cocozza S. (2004). Real time PCR quantification of frataxin mRNA in the peripheral blood leucocytes of Friedreich ataxia patients and carriers. J. Neurol. Neurosurg. Psychiatry.

[49] Santos M.M., Miranda C.J., Levy J.E., Montross L.K., Cossée M., Sequeiros J., Andrews N., Koenig M., Pandolfo M. (2003). Iron metabolism in mice with partial frataxin deficiency. Cerebellum.

[50] Coppola G., Choi S.H., Santos M.M., Miranda C.J., Tentler D., Wexler E.M., Pandolfo M., Geschwind D.H. (2006). Gene expression profiling in frataxin deficient mice: Microarray evidence for significant expression changes without detectable neurodegeneration. Neurobiol. Dis.

[51] Coppola G., Marmolino D., Lu D., Wang Q., Cnop M., Rai M., Acquaviva F., Cocozza S., Pandolfo M., Geschwind D.H. (2009). Functional genomic analysis of frataxin deficiency reveals tissue-specific alterations and identifies the PPARγ pathway as a therapeutic target in Friedreich’s ataxia. Hum. Mol. Genet.

[52] Coppola G., Burnett R., Perlman S., Versano R., Gao F., Plasterer H., Rai M., Saccá F., Filla A., Lynch D.R. (2011). A gene expression phenotype in lymphocytes from Friedreich ataxia patients. Ann. Neurol.

[53] Silva J.M., Li M.Z., Chang K., Ge W., Golding M.C., Rickles R.J., Siolas D., Hu G., Paddison P.J., Schlabach M.R. (2005). Second-generation shRNA libraries covering the mouse and human genomes. Nat. Genet.

[54] Passarelli C., Tozzi G., Pastore A., Bertini E., Piemonte F. (2010). GSSG-mediated complex I defect in isolated cardiac mitochondria. Int. J. Mol. Med.

[55] Pastore A., Piemonte F., Locatelli M., lo Russo A., Gaeta L.M., Tozzi G., Federici G. (2001). Determination of blood total, reduced, and oxidized glutathione in pediatric subjects. Clin. Chem.

[56] Nijtmans L.G., Henderson N.S., Holt I.J. (2002). Blue Native electrophoresis to study mitochondrial and other protein complexes. Methods.

[57] Eigentler A., Boesch S., Schneider R., Dechant G., Nat R. (2013). Induced pluripotent stem cells from friedreich ataxia patients fail to upregulate frataxin during in vitro differentiation to peripheral sensory neurons. Stem Cells Dev.

[58] Sandi C., Sandi M., Jassal H., Ezzatizadeh V., Anjomani-Virmouni S., Al-Mahdawi S., Pook M.A. (2014). Generation and characterisation of Friedreich ataxia YG8R mouse fibroblast and neural stem cell models. PLoS One.

